# Incidence of Nonfatal Traumatic Brain Injury–Related Hospitalizations — United States, 2018

**DOI:** 10.15585/mmwr.mm7048a3

**Published:** 2021-12-03

**Authors:** Alexis B. Peterson, Karen E. Thomas

**Affiliations:** 1 Division of Injury Prevention, National Center for Injury Prevention and Control, CDC.

Traumatic brain injury (TBI), which can disrupt normal brain function and result in short- and long-term adverse clinical outcomes, including disability and death, is preventable. To describe the 2018 incidence of nonfatal TBI-related hospitalizations in the United States by sociodemographic characteristics, injury intent, and mechanism of injury, CDC analyzed data from the Healthcare Cost and Utilization Project (HCUP) National (Nationwide) Inpatient Sample. During 2018, there were 223,050 nonfatal TBI-related hospitalizations; rates among persons aged ≥75 years were approximately three times higher than those among persons aged 65–74 years, and the age-adjusted rate among males was approximately double that among females. Unintentional falls were the most common mechanism of injury leading to nonfatal TBI-related hospitalization, followed by motor vehicle crashes. Proper and consistent use of recommended restraints (i.e., seatbelts, car seats, and booster seats) and, particularly for persons aged ≥75 years, learning about individual fall risk from health care providers are two steps the public can take to prevent the most common injuries leading to nonfatal TBIs. The findings in this report could be used by public health officials and clinicians to identify priority areas for prevention programs.

Estimates for nonfatal TBI-related hospitalizations were obtained from the 2018 HCUP National Inpatient Sample files. The National Inpatient Sample is a stratified sample of approximately 20% of hospital discharges in the United States and is sponsored by the Agency for Healthcare Research and Quality. Records were included if the primary diagnosis was an injury and a TBI-related *International Classification of Diseases, Tenth Revision, Clinical Modification* (ICD-10-CM) code (S02.0, S02.1–, S02.80X–S02.82X, S02.91, S04.02, S04.03–, S04.04–, S06–, S07.1, and T74.4) was present in any diagnosis field. A record could potentially include multiple external cause of injury codes; injury mechanism/intent categories were based on the first cause code found, as it was considered the first valid external cause of injury code. ICD-10-CM codes and more detailed methods are available online (*1*). Rates were calculated using bridged race population estimates obtained from the National Center for Health Statistics as denominators. Nonfatal hospitalizations were weighted to provide national estimates, and 95% CIs were calculated using complex survey procedures in SAS (version 9.4; SAS Institute). Age-adjusted rates were calculated using the direct method and the 2000 U.S. Census Bureau standard population. This activity was reviewed by CDC and was conducted consistent with applicable federal law and CDC policy.*

In 2018, there were 223,050 nonfatal TBI-related hospitalizations in the United States. Among nonfatal TBI-related hospitalizations with known age, 16,480 (7.4%) occurred among infants, children, and adolescents aged 0–17 years, and 70,445 (31.6%) occurred among adults aged ≥75 years (Table 1). National rates of nonfatal TBI-related hospitalizations were highest among persons aged ≥75 years (321.4 per 100,000 population) and among males (81.3 per 100,000 population, age-adjusted). The rate of nonfatal TBI-related hospitalizations among persons aged ≥75 years was approximately three times higher than that among those aged 65–74 years (105.5 per 100,000 population), and the rate among males was approximately double that among females (44.4 per 100,000 population, age-adjusted). Age-adjusted rates of nonfatal TBI-related hospitalizations were similar among non-Hispanic White persons (59.0 per 100,000 population), non-Hispanic Black persons (60.0 per 100,000 population), and Hispanic persons (59.6 per 100,000).

**TABLE 1:** Weighted estimated number* and rate^†^ of nonfatal traumatic brain injury–related hospitalizations^§^ (N = 223,050), by selected sociodemographic characteristics — National Inpatient Sample, Healthcare Cost and Utilization Project, United States, 2018

Characteristic	No.		Rate^†^ (95% CI)	
	Crude*	Adjusted^¶^	Crude*	Adjusted^¶^
**Age group, yrs (% of adjusted total)**				
0–17	16,480 (7.4)	—	22.5 (19.7–25.2)	—
0–4	6,540 (3.0)	—	33.1 (28.1–38.1)	—
5–9	2,415 (1.1)	—	12.0 (10.0–13.9)	—
10–14	3,190 (1.4)	—	15.3 (12.9–17.7)	—
15–24	19,850 (8.9)	—	46.3 (42.7–49.9)	—
25–34	21,010 (9.4)	—	46.1 (42.3–49.8)	—
35–44	17,745 (8.0)	—	43.1 (39.6–46.6)	—
45–54	21,115 (9.5)	—	50.8 (47.2–54.3)	—
55–64	28,610 (12.8)	—	67.8 (63.7–71.8)	—
65–74	32,115 (14.4)	—	105.5 (100.0–110.9)	—
≥75	70,445 (31.6)	—	321.4 (306.0–336.8)	—
**Sex**				
Male	134,650	134,635	83.7 (79.0–88.4)	81.3 (79.3–83.2)
Female	88,380	88,380	53.3 (50.7–55.9)	44.4 (43.3–45.5)
Race/Ethnicity**				
White, non-Hispanic	145,350	145,340	72.3 (68.3–76.3)	59.0 (57.5–60.4)
Black, non-Hispanic	25,195	25,195	58.7 (52.6–64.9)	60.0 (57.2–62.8)
Hispanic	28,550	28,545	47.9 (42.3–53.4)	59.6 (56.5–62.7)
Other	17,490	17,490	75.7 (68.1–83.2)	77.8 (74.0–81.6)
Unknown	6,465	6,465	—	—
**Urbanization of patient's residence**				
Large central metro^††^	72,630	72,630	72.2 (65.0–79.3)	69.1 (66.4–71.9)
Large fringe metro^§§^	51,345	51,345	62.7 (55.9–69.5)	57.4 (54.8–59.9)
Medium metro^¶¶^	49,640	49,635	72.6 (63.8–81.4)	65.8 (62.7–68.8)
Small metro***	19,400	19,400	65.2 (57.1–73.3)	57.8 (54.8–60.9)
Micropolitan (nonmetro)^†††^	16,095	16,095	59.0 (52.6–65.5)	52.5 (49.8–55.2)
Noncore (nonmetro)^§§§^	11,370	11,370	60.5 (53.9–67.1)	53.7 (50.6–56.7)
Unknown	2,570	2,560	—	—
**Total**	**223,050**	**223,035**	**68.3 (64.7–71.8)**	**62.4 (61.0–63.7)**

* Hospitalizations with missing/unknown age were included.

^†^ All rates are per 100,000 population.

^§^ In-hospital deaths (20,515) and patients who transferred from another hospital (45,205) were excluded.

^¶^ Hospitalizations with missing/unknown age were excluded. Rates were age-adjusted using the direct method to the 2000 U.S. Census Bureau standard population.

** Other includes non-Hispanic Asian, non-Hispanic Native Hawaiian or Other Pacific Islander, non-Hispanic American Indian or Alaska Native, and non-Hispanic Other.

^††^ Central counties of metropolitan areas of ≥1 million population.

^§§^ Fringe counties of metropolitan areas of ≥1 million population.

^¶¶^ Counties in metropolitan areas of 250,000–999,999 population.

*** Counties in metropolitan areas of 50,000–249,999 population.

^†††^ Micropolitan counties.

^§§§^ Not metropolitan or micropolitan counties.

In 2018, approximately 75% of nonfatal TBI-related hospitalizations were caused by either unintentional falls (51.0%) or motor vehicle crashes (23.8%) (Table 2). Rates for nonfatal TBI-related hospitalizations attributable to unintentional falls were highest among adults aged ≥75 years (263.3 per 100,000 population), 65–74 years (69.9 per 100,000 population), and 55–64 years (33.2 per population). Among all age groups, the highest rates of motor vehicle crashes leading to a nonfatal TBI-related hospitalization were among persons aged 15–24 years (24.6 per 100,000 population) and aged 25–34 years (21.9 per 100,000 population). Among the major examined unintentional and intentional mechanisms of injuries that contributed to a nonfatal TBI-related hospitalization (e.g., motor vehicle crashes, falls, being struck by or against an object, self-harm, and assault), higher total estimates and age-adjusted rates were observed among males compared with females for all mechanisms of injury (Table 3).

**TABLE 2:** Weighted estimated number and rate* of nonfatal traumatic brain injury–related hospitalizations^†^ (N = 223,050), by age group and mechanism of injury — National Inpatient Sample, Healthcare Cost and Utilization Project, United States, 2018

Age group, yrs	Mechanism of injury						
	Motor vehicle crashes	Unintentional falls^§^	Unintentionally struck by or against an object	Other or unspecified unintentional injury	Intentional self-harm^¶^	Assault	Other**
**0–4**							
No. (row %)	655 (10.0)	3,365 (51.5)	180 (2.8)	605 (9.3)	—^¶^	1,135 (17.4)	600 (9.2)
Rate (95% CI)	3.3 (2.6–4.1)	17.0 (14.4–19.6)	0.9 (0.6–1.3)	3.1 (2.4–3.8)	—^¶^	5.7 (4.3–7.2)	3.0 (1.7–4.4)
**5–9**							
No. (row %)	870 (36.4)	690 (28.9)	140 (5.9)	385 (16.1)	—^¶^	**—^††^**	305 (12.8)
Rate (95% CI)	4.3 (3.4–5.3)	3.4 (2.7–4.2)	0.7 (0.4–0.9)	1.9 (1.4–2.4)	—^¶^	**—^††^**	1.5 (0.7–2.3)
**10–14**							
No. (row %)	1,160 (37.3)	545 (17.5)	290 (9.3)	745 (24.0)	**—^††^**	**—^††^**	370 (11.9)
Rate (95% CI)	5.6 (4.5–6.7)	2.6 (2.0–3.3)	1.4 (1.0–1.8)	3.6 (2.8–4.3)	**—^††^**	**—^††^**	1.8 (1.1–2.5)
**15–24**							
No. (row %)	10,550 (53.1)	2,330 (11.7)	590 (3.0)	2,670 (13.5)	275 (1.4)	1,795 (9.0)	1,640 (8.3)
Rate (95% CI)	24.6 (22.3–26.9)	5.4 (4.8–6.1)	1.4 (1.1–1.7)	6.2 (5.5–6.9)	0.6 (0.5–0.8)	4.2 (3.7–4.7)	3.8 (2.9–4.7)
**25–34**							
No. (row %)	10,000 (47.6)	3,130 (14.9)	460 (2.2)	2,550 (12.1)	295 (1.4)	2,945 (14.0)	1,630 (7.8)
Rate (95% CI)	21.9 (19.8–24.1)	6.9 (6.1–7.6)	1.0 (0.8–1.2)	5.6 (4.9–6.2)	0.6 (0.5–0.8)	6.5 (5.7–7.2)	3.6 (2.6–4.5)
**35–44**							
No. (row %)	7,170 (40.4)	3,925 (22.1)	445 (2.5)	2,010 (11.3)	215 (1.2)	2,470 (13.9)	1,510 (8.5)
Rate (95% CI)	17.4 (15.5–19.3)	9.5 (8.7–10.4)	1.1 (0.9–1.3)	4.9 (4.3–5.5)	0.5 (0.4–0.7)	6.0 (5.3–6.7)	3.7 (2.7–4.6)
**45–54**							
No. (row %)	7,035 (33.3)	6,745 (31.9)	585 (2.8)	2,510 (11.9)	210 (1.0)	2,185 (10.3)	1,845 (8.7)
Rate (95% CI)	16.9 (15.3–18.6)	16.2 (15.0–17.5)	1.4 (1.1–1.7)	6.0 (5.4–6.7)	0.5 (0.3–0.7)	5.3 (4.6–5.9)	4.4 (3.4–5.5)
**55–64**							
No. (row %)	6,840 (23.9)	14,005 (49.0)	725 (2.5)	2,615 (9.1)	165 (0.6)	1,680 (5.9)	2,580 (9.0)
Rate (95% CI)	16.2 (14.7–17.7)	33.2 (31.2–35.2)	1.7 (1.4–2.0)	6.2 (5.6–6.8)	0.4 (0.2–0.5)	4.0 (3.4–4.5)	6.1 (4.8–7.4)
**65–74**							
No. (row %)	4,755 (14.8)	21,280 (66.3)	655 (2.0)	1,750 (5.4)	100 (0.3)	595 (1.9)	2,980 (9.3)
Rate (95% CI)	15.6 (14.1–17.1)	69.9 (66.1–73.7)	2.2 (1.8–2.5)	5.7 (5.1–6.4)	0.3 (0.2–0.5)	2.0 (1.6–2.3)	9.8 (8.2–11.4)
**≥75**							
No. (row %)	3,970 (5.6)	57,720 (82.0)	1,285 (1.8)	1,445 (2.1)	—^††^	290 (0.4)	5,680 (8.1)
Rate (95% CI)	18.1 (16.3–20.0)	263.3 (250.2–276.4)	5.9 (5.1–6.7)	6.6 (5.7–7.4)	—^††^	1.3 (1.0–1.7)	25.9 (21.4–30.4)
**Total^§§^**							
**No. (row %)**	**53,015 (23.8)**	**113,740 (51.0)**	**5,355 (2.4)**	**17,285 (7.7)**	**1,320 (0.6)**	**13,195 (5.9)**	**19,140 (8.6)**
**Rate (95% CI)**	**16.2 (14.9–17.6)**	**34.8 (33.2–36.4)**	**1.6 (1.5–1.8)**	**5.3 (4.9–5.7)**	**0.5 (0.4–0.5)**	**4.0 (3.7–4.4)**	**5.9 (4.8–6.9)**
**Adjusted^¶¶^**							
No. (row %)	53,005 (23.8)	113,735 (51.0)	5,355 (2.4)	17,285 (7.7)	1,320 (0.6)	13,195 (5.9)	19,140 (8.6)
Rate (95% CI)	16.0 (15.4–16.5)	29.8 (29.1–30.5)	1.5 (1.4–1.6)	5.2 (4.9–5.4)	0.5 (0.4–0.5)	4.1 (3.9–4.3)	5.4 (5.0–5.7)

**Abbreviation**: TBI = traumatic brain injury.

* All rates are per 100,000 population.

^†^ In-hospital deaths and patients who transferred from another hospital were excluded.

^§^ Excluded falls of undetermined intent.

^¶^ Injuries in persons aged <10 years were excluded because determining intent in younger children can be difficult. Rates for nonfatal TBI-related hospitalizations because of intentional self-harm were age-adjusted to the population aged ≥10 years.

** Includes undetermined intent, legal intervention, war, intentional self-harm for age <10 years, and cases without information about cause of injury.

^††^ Entry suppressed because of data confidentiality concerns associated with unweighted case counts ≤10.

**^§§^** Hospitalizations with missing/unknown age were included.

^¶¶^ Hospitalizations with missing/unknown age were excluded. Rates were age-adjusted using the direct method to the 2000 U.S. Census Bureau standard population.

**TABLE 3:** Weighted estimated number and age-adjusted rate* of nonfatal traumatic brain injury–related hospitalizations^†^ (N = 223,035), by sex and mechanism of injury — National Inpatient Sample, Healthcare Cost and Utilization Project, United States, 2018

Sex	Mechanism of injury						
	Motor vehicle crashes	Unintentional falls^§^	Unintentionally struck by or against an object	Other or unspecified unintentional injury	Intentional self-harm^¶^	Assault	Other**
**Male**							
No. (row %)	34,660 (25.7)	60,345 (44.8)	3,560 (2.6)	12,440 (9.2)	970 (0.7)	10,615 (7.9)	12,045 (8.9)
Rate* (95% CI)	21.2 (20.4–22.0)	35.9 (34.9–36.8)	2.2 (2.0–2.3)	7.6 (7.2–7.9)	0.7 (0.6–0.8)	6.6 (6.2–7.0)	7.3 (6.7–7.8)
**Female**							
No. (row %)	18,335 (20.7)	53,390 (60.4)	1,790 (2.0)	4,840 (5.5)	350 (0.4)	2,580 (2.9)	7,095 (8.0)
Rate* (95% CI)	10.8 (10.3–11.3)	24.4 (23.7–25.1)	0.9 (0.8–1.1)	2.8 (2.6–3.0)	0.2 (0.2–0.3)	1.6 (1.5–1.8)	3.6 (3.3–3.9)
**Total**							
**No. (row %)**	**53,005 (23.8)**	**113,735 (51.0)**	**5,355 (2.4)**	**17,285 (7.7)**	**1,320 (0.6)**	**13,195 (5.9)**	**19,140 (8.6)**
Rate* (95% CI)	**16.0 (15.4–16.5)**	**29.8 (29.1–30.5)**	**1.5 (1.4–1.6)**	**5.2 (4.9–5.4)**	**0.5 (0.4–0.5)**	**4.1 (3.9–4.3)**	**5.4 (5.0–5.7)**

**Abbreviation**: TBI = traumatic brain injury.

* Hospitalizations with missing age were excluded. Rates were age-adjusted using the direct method to the 2000 U.S. Census Bureau standard population (per 100,000 population).

^†^ In-hospital deaths and patients who transferred from another hospital were excluded.

**^§^** Falls of undetermined intent were not included.

**^¶^** Injuries in persons aged <10 years were excluded because determining intent in younger children can be difficult. Rates for nonfatal TBI-related hospitalizations because of intentional self-harm were age-adjusted to the population aged ≥10 years.

** Includes undetermined intent, legal intervention, war, intentional self-harm for those aged <10 years, and cases without information about cause of injury.

## Discussion

Nationally, 223,050 nonfatal TBI-related hospitalizations occurred during 2018. Rates varied by age group, sex, principal mechanism of injury and, within each principal mechanism of injury, by age group. Consistent with findings from a previous CDC surveillance report (*1*), the highest estimates and rates of nonfatal TBI-related hospitalizations occurred among adults aged ≥75 years and among males, and unintentional falls and motor vehicle crashes were the most common mechanisms of nonfatal TBI-related injury.

The highest rate of nonfatal TBI-related hospitalizations was among persons aged ≥75 years, the oldest age group in this study; hospitalizations among this age group can be complicated by the presence of underlying medical conditions, including hypertension, diabetes mellitus, or coronary heart disease (*2*). Older age is a known major risk factor for TBI (*2*), and following a TBI, older adults perform worse on measured cognitive abilities (e.g., naming and vocabulary) when compared with older adults without a history of TBI (*3*). Consistent with previous data suggesting that males are more likely than are females in the general adult population to sustain a TBI (*4*), this study found higher age-adjusted rates of nonfatal TBI-related hospitalizations among males compared with females across all mechanisms of injury. Reported incidence of TBI by sex is complex and potentially affected by several factors, including differing biologic vulnerabilities to injury and sex differences in care-seeking behavior (*5*).

Unintentional fall was the leading mechanism of injury contributing to a nonfatal TBI diagnosis for which the patient was hospitalized. During 2018, the highest rate of nonfatal TBI-related hospitalization attributable to falls was among adults aged ≥75 years, consistent with older age being a major risk factor for falls (*6*). Health care providers should evaluate older adult patients for signs and symptoms of TBI if they have fallen or had a fall-related injury, such as a hip fracture (*7*). Further, more older adults receive aspirin and anticoagulant therapies (e.g., warfarin [Coumadin] and non-vitamin K oral anticoagulants) as part of routine management of chronic conditions. The prevalence of anticoagulant use in this population can result in an increased likelihood of intracranial hemorrhage (*8*) and further complications from TBIs. Consistent with previous epidemiologic data (*1*), the age-adjusted rate for nonfatal TBI-related hospitalization attributable to falls was higher among males than among females. This finding might be related to circumstances of the fall, such as a larger proportion of males falling from heights (e.g., ladders) (*9*), which are more likely to result in moderate to severe injuries, including TBI.

The second most common mechanism of injury among all age groups was motor vehicle crashes, with the age-adjusted rate among males being approximately double that among females. Males are involved in more motor vehicle crash fatalities when compared with females,^†^ and data from one state suggest that this finding might be the result of a higher incidence of speeding and loss-of-control crashes among males (*10*). The likelihood of nonfatal TBI-related hospitalization from a motor vehicle crash can be reduced for persons of all ages, including older adults, by consistently and properly wearing a seatbelt while driving or riding in a motor vehicle, never driving under the influence of drugs or alcohol, and driving at recommended speeds. Consistently and properly buckling children into age- and weight/height-appropriate car or booster seats^§^ can prevent pediatric TBIs caused by a motor vehicle crash. New adolescent and young adult drivers can help prevent TBIs attributed to motor vehicle crashes by engaging in graduated driving licensing systems that help build driving skills (e.g., lane merging, passing, and maintaining a safe distance while driving) and limiting driving under high-risk conditions. Motorcyclists and bicyclists can reduce the likelihood of TBI during a crash by properly and consistently wearing a helmet. 

The findings in this report are subject to at least three limitations. First, persons who only sought care in the emergency department or outside the hospital setting (e.g., urgent care, primary care, and specialty care), who received a TBI diagnosis in federal, military, or Veterans Administration hospitals, or who did not seek care at all were not included. Therefore, this report is not a complete accounting of all nonfatal TBIs in the United States. Second, this analysis did not differentiate nonfatal TBI cases by severity of injury. Finally, the mechanism and intent of injury were unknown for 8.4% of nonfatal TBI-related hospitalizations, and as a result, estimates by mechanism of injury and injury intent are undercounts.

A TBI can happen to anyone at any age; during 2018, the oldest age group (aged ≥75 years) experienced the highest numbers and rates of nonfatal TBI-related hospitalizations. Among all nonfatal TBI-related hospitalizations, unintentional falls were the leading cause of injury, with half of these hospitalizations occurring among older adults, highlighting the need to intensify prevention efforts for falls, particularly among this age group. The CDC’s Stopping Elderly Accidents, Deaths, and Injuries (STEADI)^¶^ initiative can support health care providers in screening for fall risk, assessing modifiable risk factors, and intervening to reduce risk by updating patients’ personalized fall prevention plans. Proper restraint use (i.e., seatbelts, car seats, and booster seats) is a proven strategy for reducing motor vehicle occupant injuries, including TBIs. The CDC’s Motor Vehicle Prioritizing Interventions and Cost Calculator for States (MV PICCS)** can aid states in identifying strategies that could effectively reduce motor vehicle crash injuries. TBIs are preventable. The findings in this report could be used by public health officials to support identification of priority areas for TBI prevention programs and groups at increased risk for TBI.

**Summary**

**What is already known about the topic?**

Traumatic brain injury (TBI), an injury that can disrupt normal brain function, contributes to a substantial number of hospitalizations each year.

**What is added by this report?**

During 2018, there were 223,050 nonfatal TBI-related hospitalizations in the United States. Rates were highest among males and persons aged ≥75 years. Unintentional falls and motor vehicle crashes were the most common injuries leading to a nonfatal TBI-related hospitalization.

**What are the implications for public health practice?**

Proper and consistent restraint use (i.e., seatbelts, car seats, and booster seats) and learning about individual fall risk from health care providers are two steps the public can take to prevent the most common injuries leading to a nonfatal TBI.

## Footnotes

* 45 C.F.R. part 46.102(l)(2), 21 C.F.R. part 56; 42 U.S.C. Sect. 241(d); 5 U.S.C. Sect. 552a; 44 U.S.C. Sect. 3501 et seq.

†
https://cdan.dot.gov/query

§
https://www.cdc.gov/transportationsafety/child_passenger_safety/

¶
https://www.cdc.gov/steadi/

**
https://www.cdc.gov/transportationsafety/calculator/index.html
